# Histological Evidence for Therapeutic Induction of Angiogenesis Using Mast Cells and Platelet-Rich Plasma within A Bioengineered Scaffold following Rat Hindlimb Ischemia 

**DOI:** 10.22074/cellj.2020.6287

**Published:** 2019-07-31

**Authors:** Ali Karimi, Rasoul Shahrooz, Rahim Hobbenagh, Rahim Mohammadi, Nowruz Delirezh, Saeede Amani, Johan Garssen, Esmaeil Mortaz, Ian M Adcock

**Affiliations:** 1 Department of Basic Science, Faculty of Veterinary Medicine, Urmia University, Urmia, Iran; 2 Department of Pathobiology, Faculty of Veterinary Medicine, Urmia University, Urmia, Iran; 3 Department of Surgery and Diagnostic Imaging, Faculty of Veterinary Medicine, Urmia University, Urmia, Iran; 4 Division of Pharmacology, Utrecht Institute for Pharmaceutical Sciences, Faculty of Science, Utrecht University, Utrecht, Netherlands; 5 Nutricia Research Centre for Specialized Nutrition, Utrecht, Netherlands; 6 Department of Immunology, School of Medicine, Shahid Beheshti University of Medical Sciences, Tehran, Iran; 7 Clinical Tuberculosis and Epidemiology Research Center, National Research Institute for Tuberculosis and Lung Disease (NRITLD), Shahid Beheshti University of Medical Sciences, Tehran, Iran; 8 Cell and Molecular Biology Group, Airways Disease Section, Faculty of Medicine, National Heart and Lung Institute, Imperial College London, London, United Kingdom; 9 Priority Research Centre for Asthma and Respiratory Disease, Hunter Medical Research Institute, University of Newcastle, Newcastle, NSW, Australia

**Keywords:** Chitosan, Histology, Ischemia, Mast Cells, Platelet-Rich Plasma

## Abstract

**Objective:**

Peripheral arterial disease results from obstructed blood flow in arteries and increases the risk of amputation in acute cases. Therapeutic angiogenesis using bioengineered tissues composed of a chitosan scaffold that was enriched with mast cells (MCs) and/or platelet-rich plasma (PRP) was used to assess the formation of vascular networks and subsequently improved the functional recovery following hindlimb ischemia. This study aimed to find an optimal approach for restoring local vascularization.

**Materials and Methods:**

In this experimental study, thirty rats were randomly divided into six experimental groups: a. Ischemic control group with right femoral artery transection, b. Ischemia with phosphate-buffered saline (PBS) control group, c. Ischemia with chitosan scaffold, d. Ischemia with chitosan and MCs, e. Ischemia with chitosan and PRP, and f. Ischemia with chitosan, PRP, and MCs. The left hind limbs served as non-ischemic controls. The analysis of capillary density, arterial diameter, histomorphometric analysis and immunohistochemistry at the transected locations and in gastrocnemius muscles was performed.

**Results:**

The group treated with chitosan/MC significantly increased capillary density and the mean number of large blood vessels at the site of femoral artery transection compared with other experimental groups (P<0.05). The treatment with chitosan/MC also significantly increased the muscle fiber diameter and the capillary-to-muscle fiber ratio in gastrocnemius muscles compared with all other ischemic groups (P<0.05).

**Conclusion:**

These findings suggested that chitosan and MCs together could offer a new approach for the therapeutic induction of angiogenesis in cases of peripheral arterial diseases.

## Introduction

The prevalence of peripheral artery disease (PAD), which affects approximately 200 million people globally, is increasing (1). These patients are at increased risk of acute limb ischemia (ALI), a painful event that can lead to limb loss due to inadequate angiogenesis and collateral artery ramification which may stimulate additional functional disorders (2). 

A few attempts have been made to reduce limb morbidity in prospective randomized trials in PAD (3). However, current pharmacological treatment is ineffective, and not all patients are eligible for surgical procedure (4). Therefore, developing a new treatment strategy that will reduce both the symptoms of the disease but also the underlying pathological processes is critical. Therapeutic angiogenesis provides a potential approach to improve and increase the function of ischemic tissue via stimulating blood vessel growth, enabling tissue perfusion and therefore supporting tissue regeneration and healing (5).

Pro-angiogenic approaches have been used in numerous studies investigating growth factor and cell-based therapies (2). Clinical trials have been performed to examine the effects of modulating growth factors including fibroblast growth factor (FGF), hepatocyte growth factor (HGF), and vascular endothelial growth factor (VEGF). However, the results yielded little clinical significance apart from evidence of increased vascularity (5) Cell transplantation is a novel strategy for the treatment of critical limb ischemia (CLI) and specific bone marrow cells can be targeted to the sites of ischemia, and they contribute to blood vessel regeneration (6). 

Mast cells (MC) are circulating bone marrow-derived cells found in all connective tissues and mucosal environments particularly in perivascular regions (7). MCs release various angiogenic factors including interleukin-8 (IL-8), FGF, VEGF, and transforming growth factors-a and b (TGF-a and TGF-b) (8). Many documents indicate an association between angiogenesis and the presence of MCs in body tissues. The presence of MCs near the site of capillary sprouting is one of the evidence for the association between angiogenesis and MCs (9). 

Activated MCs synthesize large amounts of inducible nitric oxide (NO) which regulates processes such as inflammation and angiogenesis (10). NO upregulates the VEGF expression and enhances viability, proliferation, migration, association with intercellular matrix and the differentiation of endothelial cells and their formation into capillaries (11). MCs are implicated in most stages of wound healing including the initiation and modulation via acute inflammation at the growth and proliferation stages, as well as in the final remodeling of the newly formed connective tissue matrix (12). Furthermore, MCs increase the proliferation and migration of mesenchymal cells in the murine heart following infarction (13). In addition, the ability of platelet-rich plasma (PRP) to stimulate tissue regeneration is thought to be due to the effects of growth factors on progenitor cell proliferation, migration, and tube formation resulting in local angiogenesis (14).

Tissue engineering combines the use of cells, biochemical factors and various materials such as extracellular matrix to construct a scaffold that enables the formation of new viable tissue (15). Chitosan, derived from chitin, has been used as a tissue engineering scaffold as it has unique biopolymer, biocompatibility, and biodegradability properties (16). We hypothesized that the combination of MCs and PRP within a chitosan scaffold would synergize the repair processes in an ischemic model. In the current study, we examined the effects of mouse xenograft MCs and allograft platelets, in comparison with tissue modeling and bioengineering, on the promotion of angiogenesis in a rat hindlimb model of local ischemia. Murine MCs were used to more closely mimic the human clinical situation where sufficient MCs are unlikely to be obtained from the patients. 

## Materials and Methods

### Study design and animals

In this experimental study, 30 healthy white male Wistar rats, weighing approximately 200-250 g, were obtained from the animal house of the Faculty of Veterinary Medicine, Urmia University, and randomly divided into six experimental groups: a. Ischemic control group (ischemia): the femoral artery of right hind limb was transected‚ the proximal branches, superficial caudal epigastric, and lateral muscular arteries and veins were also resected, b. Phosphate-buffered saline (PBS group): the transected area around the femoral artery in the ischemic animals was immersed with PBS, c. Chitosan control group (chitosan): the transected area around the femoral artery in the ischemic animals was exposed to 50 µL chitosan gel (see below), d. MC-transplanted group (MC): the transected area around the femoral artery in the ischemic animals was immersed with 50 µLchitosan gel and 106 MCs, e. PRP-transplanted group (PRP): the transected location was immersed with chitosan and 13×10^6^ platelets, and f. PRP- and MC-transplanted group (mix): the transected location was immersed with chitosan, 13×10^6^ platelets, and 10^6^ MCs. The left hindlimbs served as non-ischemic controls (17). Animals were kept in separate chambers with stable condition Including 23 ± 3°C temperature, adequate air, humidity, and a natural light cycle for a fortnight before and throughout the experimental protocol. Standard rodent laboratory water and food were freely accessible. Samples were obtained on day 21 post-surgery. All procedures were administrated according to Ethics Committee guidelines of the Urmia University, Urmia, Iran (AECVU-175-2018). 

### Surgical procedure 

Surgical procedures were carried out under the rules and regulations of the International Association of Pain Research (18). The animals were anesthetized by intraperitoneal injection of ketamine-xylazine (5% ketamine-90 mg/kg and 2% xylazine-5 mg/kg). The animals were positioned dorsally, and the feet pulled back. The femoral artery was located, and a 5 mm length was transected before the resected stumps were ligated to initiate hindlimb ischemia. 

### Histological analysis

The animals were anesthetized and euthanized on day 21, using an overdose of ketamine-xylazine, and tissue specimens were taken and fixed in 10% formaldehyde buffer solution. After tissue processing, 6 µm paraffin sections were prepared using a rotary microtome (Microm GmbH, Germany). The sections were stained with hematoxylin and eosin (H&E) for histology, Masson’s trichrome for collagen distribution, Periodic Acid-Schiff (PAS) to assess muscle glycogen, and CD31 antibody for the analysis of capillary density and vessel diameter at both the transected location and in gastrocnemius muscles. 

Tissue samples were photographed with a digital camera (Dino-Eye-AM-7023) and analyzed using the Dino Capture 2.0 software (Dino-Lite Europe, The Netherlands) for morphometric analysis. 

### Hematoxylin and eosin staining 

In brief, slides were deparaffinized with xylene and sections rehydrated using an ethanol gradient. Sections were stained in Harris’ hematoxylin for 8 minutes, washed under running tap water for 5 minutes before 3 fast dips in 1% acid alcohol to enhance differentiation. Sections were rewashed under running tap water for 1 minute and the blue stain revealed by placing in saturated lithium carbonate solution for 1 minute. The sections were washed in running water, counterstained with eosin for 5 minutes prior to examination under light microscopy. The number of capillaries and fibers were counted at 5 random 0.025 mm^2^ areas at ×1000 magnification, and their ratios were calculated. For the histomorphometric evaluation of fibers, cross-sectional muscles were photographed with a digital camera (Dino-Eye-AM-7023) and analyzed using the Dino Capture 2.0 software at 848-fold magnification. 

### Platelet preparation

Platelets were isolated from rat peripheral blood flow using differential centrifugation, as previously described (19). 

### Mouse bone marrow-derived mast cells 

Murine MCs were obtained from hematopoietic progenitor cells generated from the bone marrow of male mice modified from a method described previously (20). In brief, the marrow from femurs and tibia were removed from 6-9 week old donor animals by flushing the bone shafts repeatedly with flushing medium using a syringe and a 27-gauge needle. The suspension of bone marrow cells was centrifuged at 1500 rpm for 10 min, and 0.5×10^6^ cells/ml were cultured for 21 days. The culture medium was composed of RPMI 1640 medium (Gibco, UK) supplemented with 15% heat-inactivated fetal bovine serum, penicillin (100 IU/mL) and streptomycin (l00 μg/mL). Two mM L-glutamine, 0.1 mM nonessential amino acids, 5×10^-5^ M 2-mercaptoethanol, and 1 mM sodium pyruvate were also added to enrich the medium. Conditioned medium from pokeweed mitogen-stimulated spleen cells (PWM-SCM) was added to the enriched media to 20% (v/v), and the cells were incubated at 37-38˚C for a further 5-7 days. At this point, non-adherent cells were transferred to new flasks, containing a fresh medium. After 3-4 weeks, MC purity was assessed by toluidine blue staining and flow cytometry.

### Pokeweed mitogen-stimulated spleen cell conditioned medium (PWM-SCM) 

Mice splenocytes (2×10^6^ cells/ml) were cultured in a 75- cm2 flask in RPMI 1640 medium with 15% FBS containing 1 mM sodium pyruvate, 0.5 M 2-mercaptoethanol, 4 mM L-glutamine 100 U penicillin/0.1 mg/ml streptomycin and nonessential amino acids (0.1 mM) containing lectin (8 μg/ml) from Phytolacca americana (Pokeweed mitogen; Sigma, St. Louis MO). The culture medium was collected after 5-6 days of the culture when the color of the medium was completely yellow. The supernatant (PWM-SCM) was obtained by centrifugation at 3000 rpm for 15 minutes; then, gently filtration through a 0.2 μm filter.

### Toluidine blue staining

The purity of the MC population was determined by staining with toluidine blue (pH=2.7). Briefly, the harvested cells were centrifuged and stained for 2 minutes after fixation using Carnoy fluid. Cellular granularity was assessed by light microscopy (21). 

### Characterization of mast cells

The expression of the high-affinity IgE receptor (FceRI) and c-Kit on harvested MCs was assessed using flow cytometry. Briefly, the cells were washed with cold PBS before blocking of cell-surface Fc receptors with 2.4G2 (PharMingen, San Diego, CA, USA). Cells were incubated with fluorescein isothiocyanate (FITC)conjugated anti-mouse Fc..RI antibody (PharMingen, USA), Phycoerythrin (PE)-conjugated anti-mouse c-kit (PharMingen, USA) or matched isotype controls for 1 hour at 4°C. Cells were washed with PBS before being analyzed by flow cytometry (FACSCalibour BD, USA). Dead cells were separated during the data analysis. 

### Preparation of chitosan solution 

Chitosan solution was prepared, as previously described. In brief, 2% (w/v) chitosan was prepared by dissolving crab shell chitosan (~400kDa, 85% deacetylated) (Fluka, Sigma-Aldrich St. Louis, MO, USA) in an aqueous solution (1% v/v) of glacial acetic acid (Merck, Darmstadt, Germany) by stirring on a hot plate at 50°C for 3 hours. The product was vacuum filtered through Whatman paper No.3 to remove any undissolved particles. Glycerol (Sigma Chemical Co., St. Louis, MO, USA) was added to 30% (w/w) of the total solid weight in solution to prepare a non- brittle product. The product [chitosan (2% w/v)] was lyophilized in acetic acid and cross-linked with 5% (w/v) tri-polyphosphate to produce a sponge-like matrix (22). The jelly-like chitosan scaffolds were prepared and 50 µL implanted at the site of femoral artery transection. 

### Immunohistochemical analysis

Tissue sections were heated at 60°C, dewaxed with xylene, and rehydrated using an ethanol gradient. Endogenous peroxidase was blocked in 0.03% hydrogen peroxide for 5 minutes. Then, sections were gently washed in buffer before incubation for 15 minutes with anti-CD31 antibody (1:500 rabbit anti-mouse, Spain) to detect endothelial cells or with anti-CD34 antibody (1:5000 ab81289) as a marker of endothelial progenitor cells and blood vessel endothelial cells according to the manufacturer’s instructions (Biocare, USA). Sections were gently rinsed in washing buffer, placed in a wet chamber with streptavidin-HRP (streptavidin conjugated to horseradish peroxidase in PBS-containing, the antimicrobial agent). Sections were gently washed by the use of washing buffer and placed in a buffer dish. Diaminobenzidinesubstrate- chromogen (DAKO, Denmark) was added to tissue slides and incubated for 5 minutes. Sections were then washed and counterstained using hematoxylin for 5 seconds before being immersed 10 times in weak ammonia solution (0.037 M/L). Sections were washed with distilled water, immunohistochemically stained and visualized as a brown stain under light microscopy. 

### DNA-laddering 

DNA laddering was performed using a commercial apoptotic DNA laddering kit (Roche Diagnostics GmbH, Mannheim, Germany). DNA was separated through a 0.8% agarose gel for 60 minutes at 60 V. lPST1-digested DNA was loaded as a control for the DNA content. Gels were stained with ethidium bromide and visualized with the Gel Doc 2000 system (Bio-Rad, California). Necrosis leads to rapid non-specific cleavage of DNA which is visualized as a smear whilst apoptosis results in 100-3000 bp DNA ladders. 

### Collagen fiber density 

Using Masson’s trichrome stain, collagen fibers were visualized by light microscopy (Zeiss, Cyber-Shot, Japan) using the MEZZURE software (Image pro-vision insight software) with a ×2.4 optical zoom. Staining intensity and distribution were evaluated by pixel counting. 

### Statistics

We used the SPSS 20 software (SPSS Inc., Chicago, USA) to analyze the data. All data are expressed as the mean and standard error of the mean (mean ± SEM). One-way ANOVA was used to compare the differences between the groups followed by Bonferroni post hoc test. The P<0.05 was considered statistically significant.

## Results

**Fig.1:**
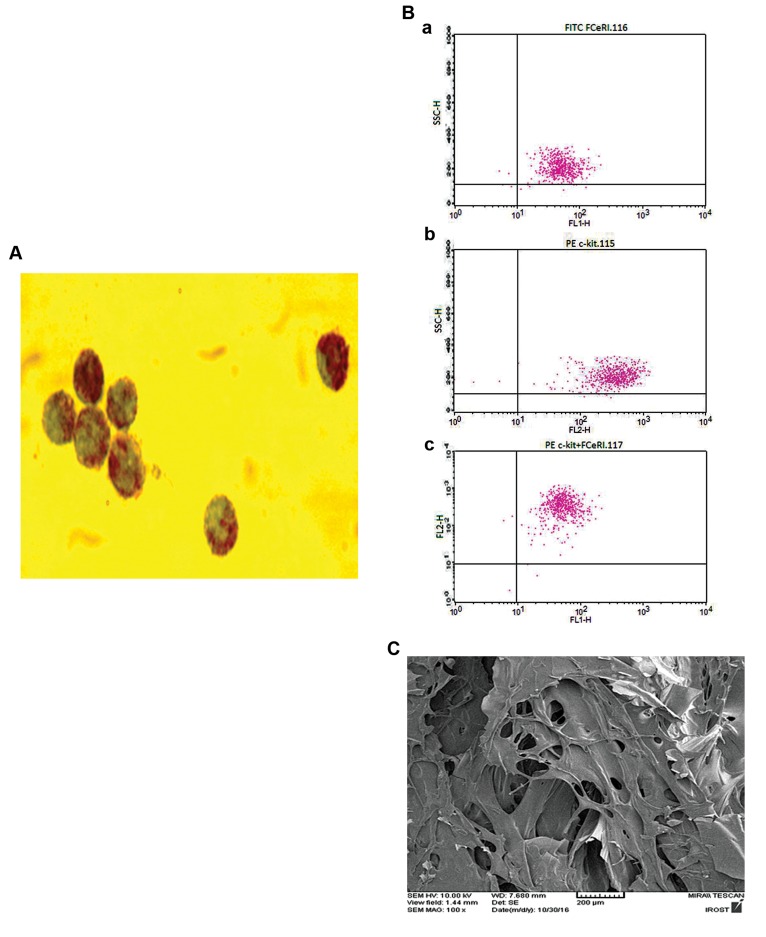
Presentation of the cell characterizations and scaffold microstructure. **A.** Murine bone marrow mast cells (BMMC) were cultured in pokeweed mitogen-stimulated spleen cell conditioned medium (PWMSCM), 20% (v/v) for 3 weeks and cells were stained with toluidine blue (representative image at ×1000 magnification), **B.** Representative flow cytometry analysis of BMMC. (a) Cells positive for FC.RI, (b) Cells positive for CD117 (c-kit), and (c) Double positive cells (92%), and **C.** Representative micrograph of scanning electron microscope to evaluate ultra-structure of porosity of the chitosan scaffold. Images are representative of at least n=3 independent experiments.

**Fig.2:**
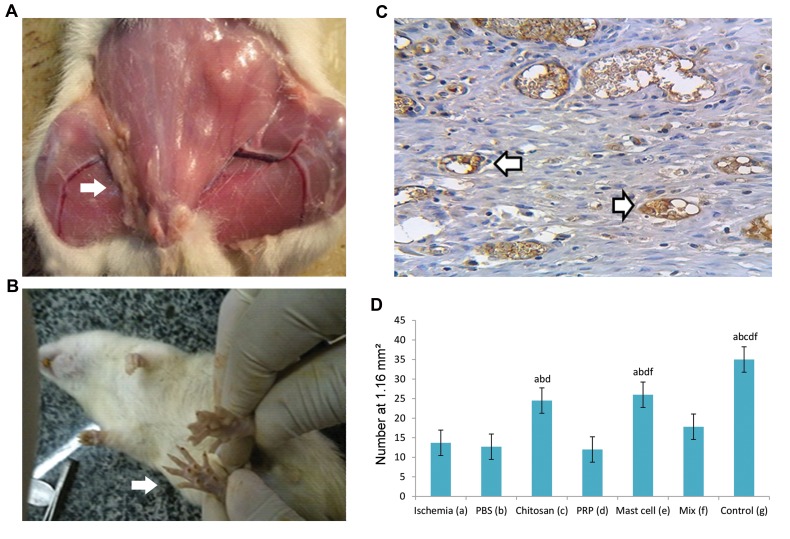
Illustrations for gross morphology of femoral artery and presentation of Immunohistochemical staining for endothelial cells and capillary density. **A.** Gross morphology of right femoral artery transection. The arrow shows the transected and transplanted area, **B.** The visual presentation of ischemiainduced necrosis of the foot pad, **C.** Immunohistochemical staining for CD31-positive endothelial cells (brownish yellow staining, arrowed) within the transplantation area (×600 magnification). Images are representative of at least n=5 independent analyses, and **D.** Bar graph shows the effect of ischemia on capillary density. All values are expressed as the mean ± SEM, a-f represent statistically significant differences (P<0.05) among indicated groups. Pictures are representative of the results from 5 animals with the left hind paw acting as a control. In this study, we used 5 animals in each group. PRP; Platelet-rich plasma, Mix; Chitosan, PRP, and mast cell group, and PBS; Phosphate-buffered saline.

After 3 weeks of cell culture, more than 92% of bone marrow cells differentiated into MCs as determined by Toluidine Blue staining (Fig .1A) and flow cytometry (Fig .1B). Scanning electron microscopy demonstrated the porosity of the chitosan scaffold (Fig .1C) On the 21^st^ day, after the operation, macroscopically visible connective tissue was present in the graft region of the cell transplantation groups (Fig .2A). In the ischemia groups, due to femoral artery transections, the evidence of necrosis was observed in the foot pads and fingers (Fig .2B). 

### Capillary density findings in the femoral artery resected area

A combination of H&E, Masson’s trichrome staining, and CD31 antibody verified the presence of endothelial cells within capillaries. Capillaries were counted at the site of femoral artery resection using an optical microscope (magnification ×400) and a graded lens (1.16 mm square mesh size) (Fig .2C). Ischemia resulted in a significant decrease in capillary density that was significantly (P<0.05) reversed in both the chitosan and the chitosan/MC groups (Fig .2D). The other treatments did not significantly enhance the capillary density compared with PBS treatment. Interestingly, the presence of PRP significantly reduced the ability of MCs to enhance capillary density (Fig .2D, comparison of MIX versus MC groups).

### Histomorphometric analysis of vessels in the femoral artery resected area

**Fig.3:**
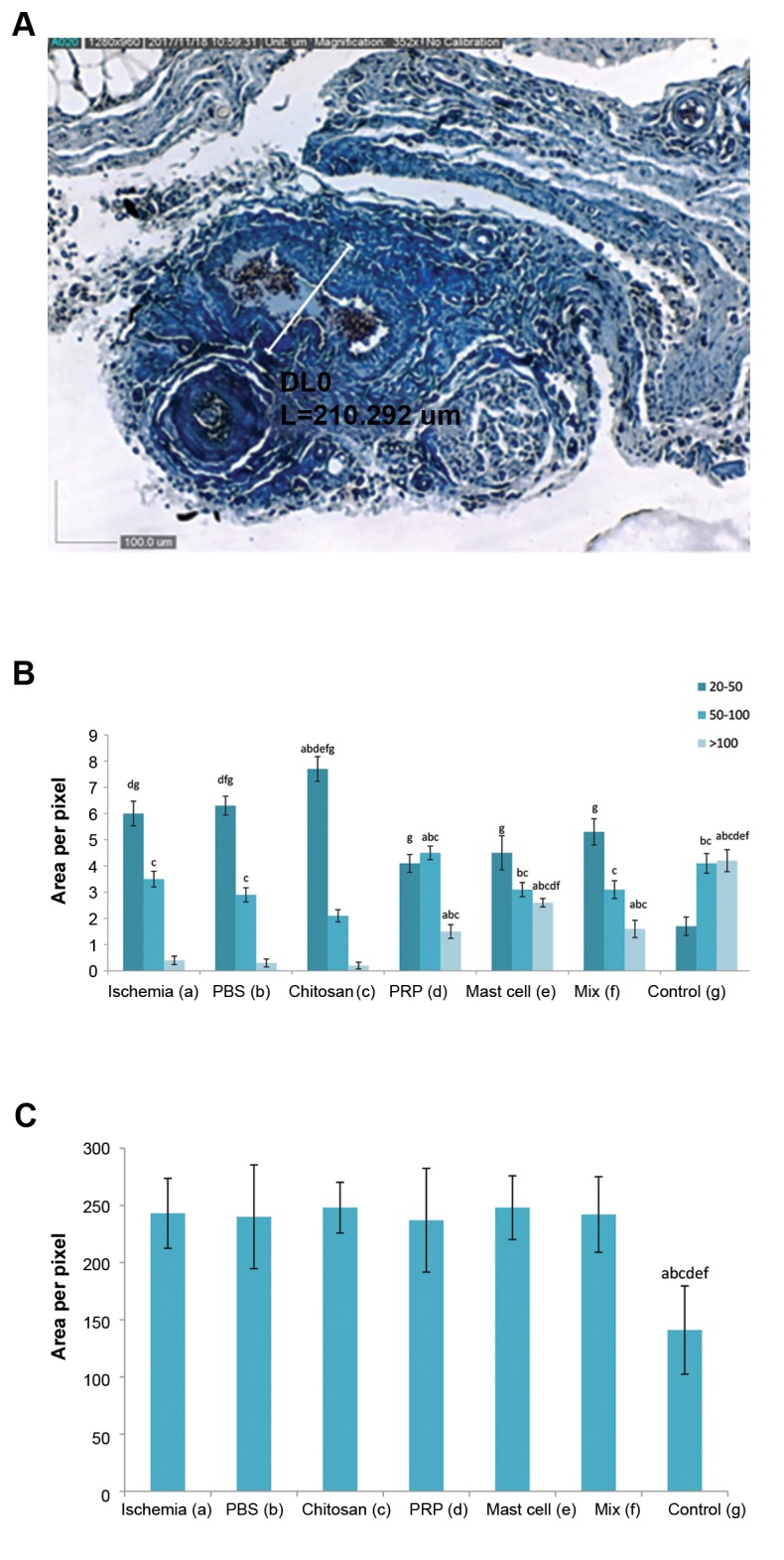
Micrograph and histograms presented collagen distribution andvessel morphometry in the cell transplanted area. **A.** Representativemicrograph (×352 magnification) showing individual morphometricanalysis of blood vessels (collagen deposition is shown as blue color) stained with Masson’s trichrome staining method and evaluated with the Dino Capture 2.0 software, **B.** The bar graph shows vesselmorphometry in experimental groups in femoral artery transected area according to cross-sectional thickness (20-50, 50-100, and >100 µm), and **C.** Histogram showed a semi-quantitative comparison of connective tissue density (intensity and distribution) Ischemiainduced distribution of collagen fibers at the site of femoral artery resection was unaffected by any intervention. All values are expressed as the mean ± SEM, a-g represent statistically significant differences (P<0.05) among indicated groups. In this study, we used 5 animals in each group. PRP; Platelet-rich plasma, Mix; Chitosan, PRP, and mast cell group, and PBS; Phosphate-buffered saline.

The histomorphometric analysis of tissue vessels was stratified into 3 groups according to the cross-sectional thickness (20-50, 50-100, and >100 μm) at the site of femoral artery transection (Fig .3A). Ischemia resulted in a significant increase in the number of small vessels (P<0.05) and a considerable reduction in the number of large vessels (P<0.05, Fig .3B). There was no significant change in the number of intermediate-sized vessels (50- 100 μM).

Chitosan alone had no effect on the ischemia-induced reduction in large vessels, increased the number of small vessels (P<0.05) and decreased the numbers of intermediate vessels (P<0.05) compared with ischemia, PBS and control animals. The number of large vessels in the chitosan/MC-treated ischemia group was significantly higher than the other groups (P<0.05) although did not reach the control levels (Fig .3B).

### Collagen fiber density in femoral artery resected area

Ischemia induced a significant increase in the distribution of collagen fibers at the site of femoral artery resection (P<0.05). No intervention affected this distribution (Fig .3C).

### Capillaries to muscle fiber ratio 

**Fig.4:**
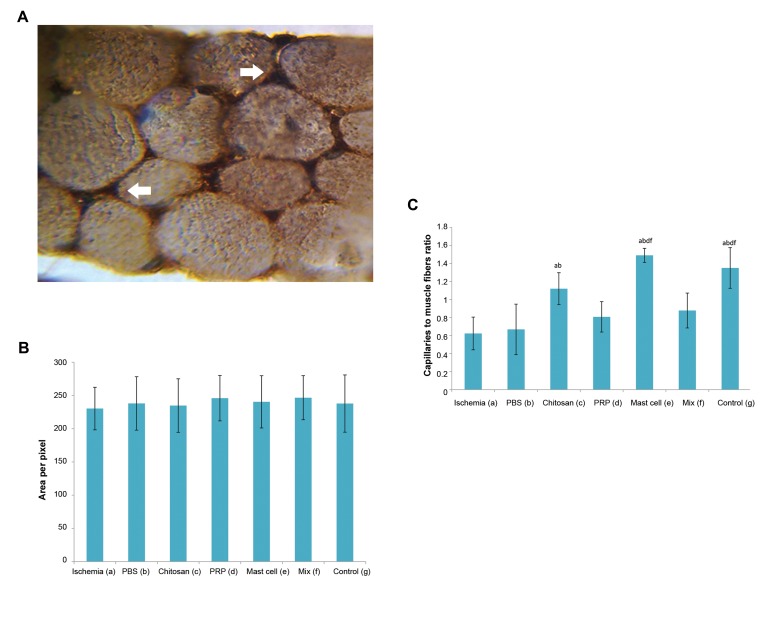
Micrograph and histogram presented the blood vessels data’s and connective tissue density in the gastrocnemius muscles. **A.** Immunohistochemical staining for CD34-positive endothelial cells (dark brown, arrowed) between the gastrocnemius muscle fibers (×1500magnification), **B.** The graph indicates semi quantitative intensity of endomysium and perimysium (connective tissue) density in different groups, and **C.** The effect of ischemia and interventions on the capillary to gastrocnemius muscle fiber ratio. All values are expressed as the mean ± SEM, a-f represent statistically significant differences (P<0.05) among indicated groups. In this study, we used 5 animals in each group. PRP; Platelet-rich plasma, Mix; Chitosan, PRP, and mast cell group, and PBS; Phosphate-buffered saline.

A combination of H&E staining, Masson’s trichrome stain and vessel endothelial cell staining (CD34+ cells) was used to count capillaries (Fig .4A). Ischemia significantly reduced the ratio of capillaries to muscle fibers at the site of femoral artery transection. This reduced ratio was significantly increased and restored to the control levels in the chitosan alone and the chitosan/MC-treated groups (P<0.05). Neither the chitosan/PRP nor chitosan/MIX groups showed significant differences when compared with the ischemia or PBS groups (Fig .4B). 

### Endomysium and perimysium (connective tissue) density in different groups 

Masson’s trichrome staining was carried out to determine the connective tissue density in the endomysium and perimysium area of gastrocnemius muscles. It demonstrated a high variability within the groups with no significant effect of ischemia or the various interventions on stained sections (Fig .4C).

### Comparison of gastrocnemius muscle fiber diameter 

**Fig.5:**
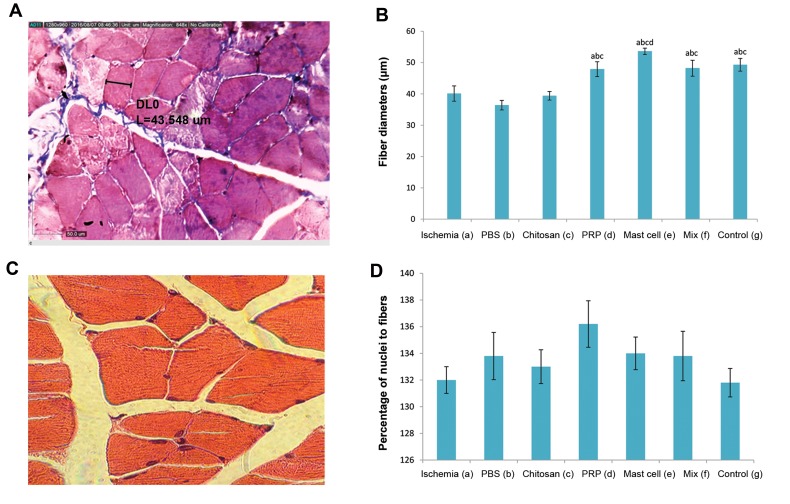
Illustrations for histomorphometric analysis of gastrocnemius muscles. **A.** Representative morphometric analysis of gastrocnemius muscle fibers (×848 magnification) stained with Masson’s trichrome stain and determined using the Dino Capture 2.0 software, **B.** Histogram showed the effect of ischemia and various interventions on fiber diameter, **C.** Representative H&E stained a transverse section of muscle demonstrating the calculation of the ratio of the nuclei to the number of muscle fibers (×1000 magnification), and **D.** Histogram showed the percentage of nuclei to muscle fibers. All values are expressed as the mean ± SEM, a-d represent statistically significant differences (P<0.05) among indicated groups. In this study, we used 5 animals in each group. PRP; Platelet-rich plasma, Mix; Chitosan, PRP, and mast cell group, PBS; Phosphate-buffered saline, and H&E; Hematoxylin and eosin.

Gastrocnemius muscle fiber diameter, as determined using the Dino Capture 2.0 software (Fig .5A), was significantly (P<0.05) reduced in the ischemia group. Chitosan alone had no effect on the muscle fiber diameter, but this parameter was significantly (P<0.05) restored to the levels of the control group in the chitosan/PRP, chitosan/MC and chitosan/MIX groups (Fig .5B).

### Estimation of the percentage of nuclei to muscle fibers 

The percentage of nuclei within muscle fibers was highly variable, and no significant effect of ischemia or any intervention was observed (Fig .5C). 

### Estimations the amount of muscle glycogen in different groups 

**Fig.6:**
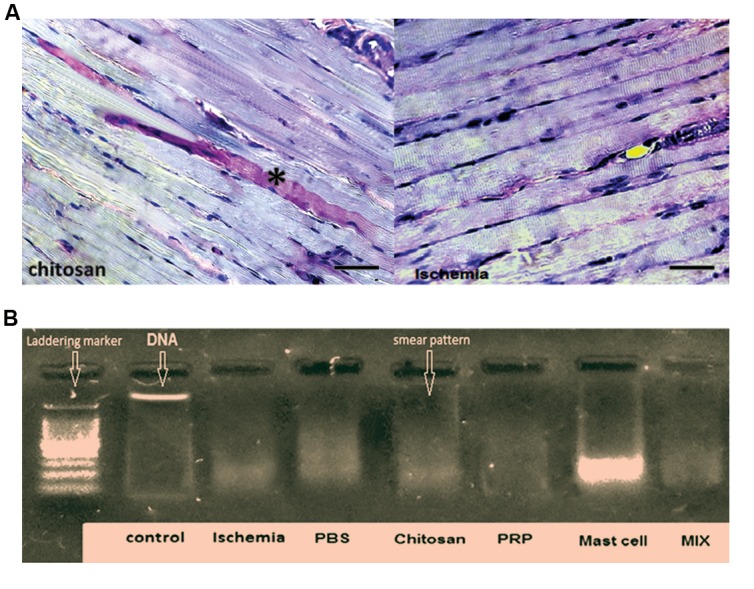
Illustration of PAS staining method and DNA fragmentation analysis of gastrocnemius muscles. **A.** Representative micrograph showing pink-red periodic acid-Schiff (PAS) staining to assess muscle glycogen. PAS staining was intense in some muscle fibers (*) but overall was nearly the same for all groups and **B.** DNA was isolated from rat muscle and prepared for the DNA fragmentation analysis. Ischemia induced DNA smearing considered a marker of necrosis, which was not affected by any of the interventions studied. The left column is a DNA marker indicating DNA “laddering,” associated with apoptosis. All sham and treated groups demonstrate a “smear” pattern, indicating the typical sign of necrosis (100-3000 bp). In this study, we used 5 animals in each group.

PAS staining indicated no difference in tissue glycogen of muscle fibers due to ischemia or following any intervention (Fig .6A). 

### Investigation of necrosis in various muscle groups 

The evaluation of DNA smearing, as a marker of necrosis, and DNA laddering, indicative of apoptosis, demonstrated the presence of ischemia-induced necrosis (smearing) rather than apoptosis (laddering) (Fig .6B). The degree of DNA smearing was not significantly affected by any of the interventions studied. 

## Discussion

Our results indicate that ischemia causes a marked reduction in capillary density and the number of large vessels, but increased the number of small vessels and the distribution of collagen fibers. These changes were associated with reduced gastrocnemius muscle fiber diameter and the capillary: gastrocnemius muscle fiber ratio. Bioengineering using a chitosan scaffold alone restored ischemia-induced capillary density and the capillary: muscle fiber ratio and further enhanced ischemia-induced small vessel formation. The combination of a chitosan scaffold and activated MCs reversed the ischemia-induced reduction in capillary density and increased the mean number of small blood vessels. This combination also enhanced the ischemiainduced reduction in large blood vessels at the site of femoral artery transection and significantly increased the muscle fiber diameter and the capillary-to-gastrocnemius muscle fiber ratio.

The pathophysiology of major artery blockade indicates that ischemia occurs when the blood flow from arteries within regions adjacent to the affected tissue is reduced, and the resulting peripheral artery expansion is insufficient to allow normal blood flow to be restored to the tissue (23). To overcome this ischemia, the microvasculature and vessels of the affected area create anastomosis leading to the formation of large blood vessels. This results in increased blood flow to the tissues overcoming the requirement for small vessels and allowing the effect of ischemia to be decreased (9). The reversal of the reduction in the capillary density in response to ischemia by all the interventions studied may result from the activation of signals released from the existing vascular tissue inducing new vessel formation (24) or factors released from platelets and MCs.

The release of MC-derived factors including VEGF, bFGF, TGF-ß, TNF-α, and IL-8 have been implicated in this increased angiogenesis (8). Previous studies have reported various roles of bFGF in the initiation of vascularization, with the modulation of endothelial cell migration, proliferation, and differentiation (25). bFGF can also stimulate the growth of large vessels in smooth muscle tissue (26). Moreover, VEGF can increase vascular permeability and vascularization (27). A limitation of the current study is the failure of analyzing the local growth factor levels. Furthermore, as this is a xerograph model, there is a potential occurrence of immunosuppression. Although unlikely to occur in the time-frame of the current experiment, future studies should look for markers of immunosuppression.

Several studies have focused on the impact of PRP on the vascularization including the role of PRP in the healing of stomach and diabetic wounds (28). In addition, the proliferation, differentiation, and migration of human microvascular endothelial cells are enhanced by PRP in *in vitro* and an *in vivo* model of neonatal mouse retinal angiogenesis (29).

We hypothesized that MCs and platelets would have a synergistic effect on neovascularization due to the ability of platelet-derived growth factor (PDGF) to enhance MC differentiation through activation of stem cell factor (SCF) receptors and MC-derived platelet-activating factor (PAF) which are able to assemble and degranulate platelets (30). However, no additive or synergistic effects on the induction of vascularization was observed in the present study. Indeed, for most of the reported outcomes, a combination of PRP and MCs had a lesser effect than MCs when used alone, indicating a degree of functional antagonism. Also, MCs and platelets induce stimulating effects on collagen synthesis and contribute to tissue fibrosis (31, 32). In our current study, ischemia induced the formation of collagen fiber which was unaffected by the presence of MCs or platelets either alone or in combination.

We investigated the effects of ischemia and potential neovascularization interventions on the gastrocnemius muscle due to a link between general blood circulation and hindlimb musculoskeletal systems. Ischemia reduced the diameter of gastrocnemius muscle fiber and capillary-to-muscle ratio without affecting the density of connective tissue in gastrocnemius endomysium and perimysium areas or the number of muscle nuclei. MCs, PRP, and mixed treatment completely reversed the decreased diameter of muscle fiber induced by ischemia with no difference between each treatment. These results were similar to those seen with micro-fractured fat tissue (Lipogems) containing human adipose-derived stem cells (hASCs) which produced greater tissue repair and reduced localized inflammation in a rat model of chronic hindlimb ischemia downstream of enhanced endothelial cell proliferation (33). Furthermore, recent studies have demonstrated that ischemia-induced gastrocnemius muscle atrophy was significantly reversed by VEGF, nerve growth factor (NGF) (34), and human smooth muscle cell transplantation (17).

Our data support the hypothesis that the formation of a local microvasculature is essential for communication between the general blood circulatory system and the lower limb circulatory system. An increase in the number of capillaries in the chitosan alone group could enhance this interaction due to the porous nature of the structural scaffold allowing angiogenesis to occur (35). Furthermore, tissue engineering experiments have indicated that human microvascular endothelial cells can drive vascularization within the host liver after implantation following Some recent surveys have shown that after transplantation, anastomosis of 25-250 µm diameter (36).

Despite the transection of the femoral artery and the induction of ischemia, our data show that gastrocnemius can maintain muscle glycogen even though the reduced blood flow would be unable to deliver the entire metabolic requirements to the affected muscles. This would indicate that a degree of metabolic reprogramming occurs in this muscle under ischemic conditions. This confirms a previous study which revealed that parallel with the increased absorption of glucose by insulin-dependent receptors (GLUT4) in hypoxic muscles, the amount of muscle glycogen was relatively constant (37). The switch from adipose to connective tissue following the ischemia may affect the overall tissue metabolic status due to the change in metabolic demands. A limitation of our study was the failure of measuring the expression of either GLUT4 or glucose uptake in specific tissue/cells types. 

The current results also indicate that the density of connective tissue (endomysium and perimysium) is not a sign of structural changes during ALI. Previous studies indicated that although the inter-myofibrillar network in the endomysium of ischemic muscle tissue was coarser than the normal, the amount of connective tissue was not significantly increased (38). The change in fiber coarseness may result from altered muscle necrosis rather than apoptosis as demonstrated by the presence of DNA smearing rather than DNA laddering. This agrees with a previous study examining ischemia and reperfusion in rat leg muscles (39). This may reflect the apoptotic resistance observed in fully matured muscle (40).

## Conclusion

These findings suggest that bioengineered tissues incorporating MCs within a chitosan scaffold could offer a new approach for therapeutic angiogenesis to improve arterial diseases. However, further research in this area is required to determine the optimal combination of scaffold and cells. 
